# Physiological Concentrations of Leptin Do Not Affect Human Neutrophils

**DOI:** 10.1371/journal.pone.0073170

**Published:** 2013-09-16

**Authors:** Vera M. Kamp, Jeroen D. Langereis, Corneli W. van Aalst, Jan A. van der Linden, Laurien H. Ulfman, Leo Koenderman

**Affiliations:** 1 Department of Respiratory Medicine, University Medical Centre Utrecht, Utrecht, The Netherlands; 2 Laboratory of Pediatric Infectious Diseases, Radboud University Medical Centre, Nijmegen, The Netherlands; University of Torino, Italy

## Abstract

Leptin is an adipokine that is thought to be important in many inflammatory diseases, and is known to influence the function of several leukocyte types. However, no clear consensus is present regarding the responsiveness of neutrophils for this adipokine. In this study a 2D DIGE proteomics approach was used as an unbiased approach to identify leptin-induced effects on neutrophils. Additionally chemotaxis and survival experiments were performed to reproduce results from literature showing putative effects of leptin on these neutrophil responses. Leptin did not induce any significant changes in the proteome provided leptin was added at physiologically relevant concentrations (250 ng). Our leptin batches were biologically active as they induced proliferation in LeptinR expressing Ba/F3 cells. At high concentrations (25000 ng) leptin induced a change in neutrophil proteome. Seventeen differently regulated spots were identified of which twelve could be characterized by mass spectrometry. Two of these identified proteins, SerpinB1 and p40 phox, were chosen for further analysis but leptin-induced expression analyzed by western blot were highly variable. Additionally leptin also induced neutrophil survival at these high concentrations. No leptin-induced chemotaxis of human neutrophils was detected at any concentration. In conclusion, physiological concentrations of leptin do not affect neutrophils. High leptin concentrations induced survival and changes in the neutrophils proteome, but this was most likely mediated by an indirect effect. However, it cannot be ruled out that the effects were mediated by a yet not-identified leptin receptor on human neutrophils.

## Introduction

Leptin is an adipokine involved in the control of energy intake but also in immunity [1]. It is a protein which has structural similarities with pro-inflammatory cytokines such as IL-6, IL-12 and granulocyte colony-stimulating factor (G-CSF) [1]. The leptin receptor can be expressed in six alternatively spliced forms [1]. From these six receptors variants the OBRb receptor is the main signaling receptor [1], [2]. The other splice variants of the receptor lack all or most of the intracellular tail that is necessary for signaling, although there are suggestions that the small intracellular tail of OBRa can signal [3]. The OBRb is expressed by several leukocytes such as monocytes, macrophages and lymphocytes [4]. This explains leptin's ability to regulate the immune system. Indeed, the importance of leptin as a cytokine has been shown in several inflammatory disease models. Leptin knockout mice show decreased survival upon *S. pneumonia* infection. Reintroducing leptin partly restored the survival [5]. Leptin has also been shown to be important for the induction of inflammation in murine models of influenza infection and cigarette smoke exposure [6], [7]. In addition, data obtained in humans demonstrate an association between leptin concentrations and inflammation in tuberculosis patients [8].

Neutrophils are important effector cells in innate immune responses and are one of the first cells to respond to tissue damage [9]. Their recruitment is influenced by leptin but there is no consensus regarding the underlying mechanism. Several models have shown that increased leptin concentrations result in a decreased neutrophil influx [7], [10]. Others have shown an increased neutrophil influx after leptin administration in *S pneumonia* induced pneumonia [11]. Also, the absence of leptin signaling in leptin receptor knockout mice resulted in decreased neutrophil recruitment in acute lung injury [12].

There is debate whether the reported effects of leptin on neutrophils are direct or indirect. It has been suggested that leptin influences the production of cytokines and glucocorticoids by other cells and, thereby, alters the neutrophil response [10], [13], [14], [15]. Other studies showed direct effects of leptin on neutrophil chemotaxis [16], [17]. Otonello and Montecucco showed leptin induced inhibition of chemotaxis towards IL-8, C5a [16] and fMLF [17], whereas leptin alone induced chemotaxis in these studies. In a murine study neutrophil migration towards KC was decreased both in db/db mice and diet induced obese mice suggesting that leptin decreased their chemotaxis capacity [12].

Apart from these chemotaxis studies little is known about direct effects of leptin on neutrophils. Rafail et al indicated that leptin stimulation of isolated neutrophils induced TNFα production by neutrophils and subsequently induced tissue factor expression [18]. Neutrophils from ob/ob mice showed decreased phagocytosis when incubated with *K. Pneumoniae in vitro*. The phagocytosis was restored when leptin was replenished [19]. This study would suggest that an increased bacterial clearance was present upon leptin stimulation. Also neutrophil survival was shown to be induced by leptin via a PI3K and P38 dependent mechanism [20]. Yet, the concentrations needed for this survival were a-physiologically high 0.5 μM (8000 ng/ml), because concentrations found in blood of lean and obese subjects are 7.5±9.3 and 31.3±24.1 ng/ml respectively [20], [21].

As only few studies described direct effects of leptin on neutrophils, and results were contradictory, we decided to reappraise the issue of leptin responsiveness of human neutrophils isolated from healthy individuals. Additionally an unbiased 2D-DIGE proteomics approach was used to define novel effects of leptin on neutrophils.

## Materials and Methods

### Reagents

Human serum albumin (HSA) and pasteurized plasma solution were purchased from the Central Laboratory of the Netherlands (Sanquin, Amsterdam, the Netherlands). Isolation buffer contained PBS supplemented with pasteurized plasma solution (10%) and trisodium citrate (0.4% (w/v)). Incubation buffer contained 20 mM HEPES, 132 mM NaCl, 6 mM KCl, 1 mM MgSO4, 1.2 mM KH2 PO4, supplemented with 5 mM glucose, 1.0 mM CaCl2, and 0.5% (w/v) HSA.

Recombinant human Leptin (R&D systems, Minneapolis, MN, USA). Human rGM-CSF was a gift from Prof. Angel Lopez (Institute of Medical and Veterinary Sciences, Adelaide, Australia). Annexin V-PE and 7AAD was purchased from Beckman D, Franklin Lakes, NJ, USA), N-formylmethionyl- leucyl-phenylalanine (fMLF) and luminol were purchased from Sigma (St. Louis, MO, USA). Pharmacological inhibitor SB203580 was purchased from Kordia Life Sciences (Leiden, The Netherlands). LY294002 was from Biomol (Plymouth Meeting, PA, USA) Mouse IL3 was produced in COS cells. All other materials were reagent grade.

### Antibodies

Anti p40 phox was purchased from novus biologicals (Littleton, CO, USA) and anti-phosphor-p40 phox (Thr154) was from cell signaling (Danvers, MA, USA). Anti β-tubulin was from Santa Cruz (Santa Cruz, CA, USA) and anti SerpinB1 was purchased from Abcam (Cambridge, UK). HRP-coupled swine anti-rabbit and rabbit anti-goat were from DAKO (Glostrup, Denmark).

### Granulocyte isolation

Human blood of healthy volunteers was collected using the internal donor service in the hospital. Collection of blood via this donor service was approved by the Ethics Committee of the University Medical Centre Utrecht and complies with the Declaration of Helsinki and the Good Clinical Practice guidelines. Healthy volunteers gave written informed consent. Cells were isolated as described previously [22]. In short, cells were separated by centrifugation over Ficoll-Paque PLUS (GE Healthcare, Uppsala, Sweden) for 20 min at 900×g. The mononuclear cell layer was removed. Erythrocytes were lysed in isotonic ice-cold NH4Cl solution followed by centrifugation at 4°C.

### Baf3 OBRb cell proliferation

Baf3 cells transfected with human OBRb were a kind gift of professor Jan Tavernier (Cytokine Receptor Laboratory, VIB – University of Gent, Gent, Belgium) [23]. Experiments were performed in RPMI culture medium supplemented with 8% fetal bovine serum, penicillin and streptomycin, all purchased from Gibco, invitrogen (Breda, the Netherlands). Proliferation at 48 h was measured by [3H]thymidine incorporation [1 μCi/well (Amersham, Little Chalfont, UK)], which was added during the last 18 h of culturing. Proliferative responses are expressed as the mean [3H]thymidine incorporation as counts per minute (c.p.m.) of triplicate wells.

### Migration assay using the Boyden chamber

Neutrophil migration was measured in the modified Boyden chamber assay as described previously [24]. In short, cellulose nitrate filters (pore width 8 μm, thickness 150 μm; Sartorius) were soaked in incubation buffer and neutrophils (25 μl of 2×10^6^/ml) were added per well and allowed to migrate for 1.5 h at 37°C. Filters were fixed, stained with hematoxylin (Weigert's method), and embedded in malinol. Analysis of the filters was performed by an image analysis system (Quantimet 570 C; Leica) and an automated microscope to score the number of cells at 15 intervals of 10 μm in the z-direction of the filters. The results are expressed as the chemotactic index, indicating the mean migrated distance (in micrometers), excluding cells with migration 0.

#### Neutrophil chemotaxis and chemokinesis in fibrin gels

Neutrophils were resuspended in incubation buffer and mixed with fibrinogen (2 mg/ml) and Thrombin (2 Units/ml), the end concentration of the cell suspension was 0.8×10^6^ cells/ml. After mixing, 30 μL of this cell suspension was pipetted into each channel of a plastic chamber slide (μ-Slide VI, ibidiTreat; Integrated BioDiagnostics (IBIDI), Munich, Germany) and the mixture was allowed to polymerize for several minutes at room temperature. Mixtures of cell suspensions and fibrinogen/thrombin were made, mixed and added to the chambers. The resulting fibrin gels have a thickness of 400 µm. The slide chamber was then transferred to an incubator hood (37°C) on a Leica DXMRE microscope and temperature equilibrated for at least 10 minutes, before multi spot time lapse imaging at 37°C was performed.

For chemotaxis analysis a similar method was used as for chemokinesis, but instead IBIDI 3D chemotaxis slides were used (μ-Slide Chemotaxis 3D, ibidiTreat; Integrated BioDiagnostics (IBIDI), Munich, Germany). Cells were suspended in fibrin gel mixtures and were pipetted in the central channels of the IBIDI 3D slide. The upper chambers were filled with leptin, positive control fMLF (10−8 M) or negative control incubation buffer. Lower compartments were always filled with incubation buffer.

#### Imaging and image analysis migration in fibrin gels

Multi-spot time-lapse imaging was performed with a computer assisted microscopy system (Quantimet for Windows (Qwin), DXMRE microscope, PL fluotar 5x low power objective lens (Leica, Heidelberg, Germany)).

Sequences consisted of 100 images per spot with a maximum of 6 revisited spots. The time-lapse interval was typically 15–25 seconds.

Images were imported into the Optimas image analysis package (Media Cybernetics, Inc. Bethesda, MD). Custom-made macros (Arithmetic Language for Images, ALI) were used to plot the migrating cells using threshold-based detection and nearest neighbor tracking. Tracks and final vectors of migrating cells were plotted and analyzed for speed, directness and directionality. Each analysis incorporated several tens to hundreds of tracked cells.

### Flow cytometry

Neutrophils were isolated and stained with antibodies for 30 min at 4°C in isolation buffer. Cells were washed before analysis on FACSCalibur (Becton Dickenson, San Jose, CA, USA). Data are expressed as mean fluorescence intensity.

### Neutrophil survival

Isolated neutrophils were incubated in RPMI with 0.5% Human serum albumin for 24 h at 37°C. After 24 hours cells were stained with Annexin V PE and 7AAD and measured by flow cytometry on a FACSCalibur (Becton Dickenson, San Jose, CA, USA). Survival is defined as the percentage Annexin V, 7AAD negative cells.

### Preparation of protein extracts

Isolated neutrophils were stimulated for 4 hours with different concentrations of leptin at 37°C. After 4 hours neutrophils were washed twice (0.34 M sucrose, 1 mM EDTA, and 10 mM Tris) and lysed in lysis buffer (10 mM Tris, pH 7.4, 10% glycerol, 1% NP40, 50 mM NaF, 20 mM tetra-Na pyrophosphate, 1 mM DTT, 2 mM vanadate, 1 mM PMSF, and 1× Complete EDTA-free protease inhibitor cocktail tablet (Roche)). Proteins were precipitated with 80% acetone and dissolved in labeling buffer (8 M Urea, 2 M Thiourea, 4% CHAPS, and 10 mM Tris pH 8.5) for CyDye labeling or IEF buffer for 2D western blots (8 M Urea, 2 M Thiourea, 4% CHAPS, 150 mM DTT, 1.0% IPG buffer 3–10 NL, and 0.002% Bromophenol blue).

### CyDye Labeling

Protein extracts were labeled using the fluorescent cyanine dyes developed for 2D-DIGE technology (GE Healthcare, Uppsala, Sweden) following manufacturer's protocol. Protein extracts (50 μg) were labeled with 400 pmol of fluorescent dye (Cy2, Cy3, or Cy5) for 30 min on ice. Protein samples were randomly labeled with Cy3 or Cy5. Internal reference sample, created by pooling 25 μg of each protein sample, was labeled with Cy2. Labeling was stopped by adding 1 μL of 10 mM lysine followed by 15 min incubation and addition of an equal volume of 2× IEF buffer (8 M Urea, 2 M Thiourea, 4% CHAPS, 300 mM DTT, 2.0% IPG buffer 3–10 NL, and 0.004% Bromophenol blue) to each sample. Cy3 sample, Cy5 sample, and Cy2 internal reference sample were pooled and brought up to a final volume of 450 μL with IEF buffer (8 M Urea, 2 M Thiourea, 4% CHAPS, 150 mM DTT, 1.0% IPG buffer 3–10 NL, and 0.002% Bromophenol blue).

### 2D Gel Electrophoresis and Analysis

Protein samples were passively rehydrated into 24 cm pH 3–10 NL strips (GE Healthcare, Uppsala, Sweden) for minimal 10 h followed by isoelectric focusing using a manifold-equipped IPGphor IEF unit (GE Healthcare, Uppsala, Sweden) according to the manufacturer's protocol. The cysteine sulfhydryls were reduced with 1.0% DTT and carbamidomethylated with 2.5% Iodoacetamide in equilibration buffer (30% glycerol, 2% SDS, 6 M urea, and 75 mM Tris, pH 8.8). Second-dimensional SDS-PAGE was performed on hand-cast 12% SDS-PAGE gels using low fluorescence glass plates. Electrophoresis was carried out at 0.2 W/gel for 2 h followed by 1 W/gel until completion using an Ettan DALT-12 unit (GE Healthcare, Uppsala, Sweden). Gels were scanned using a Typhoon 9410 imager at 100 μm resolution (GE Healthcare, Uppsala, Sweden). Scan settings were optimized for a maximal signal of 85 000 counts. Gel images were cropped using ImageQuantTL 2003 (GE Healthcare, Uppsala, Sweden), spot detection was performed with DeCyder 6.5 DIA (Difference In-gel Analysis) software (GE Healthcare, Uppsala, Sweden), and gel images were matched using DeCyder 6.5 BVA (Biological Variation Analysis) software (Healthcare, Uppsala, Sweden).

### Spot picking

A Flamingo stained preparative gel with 400 μg neutrophil lysate was matched with the DeCyder analysis program and spots were picked automated with an Ettan Spot Picker (GE Healthcare) following manufacturer's protocol.

### In gel digestion and mass spectrometry analysis

In-gel digestion and mass spectrometry analysis was outsourced to ServiceXS (Leiden, The Netherlands). Excised gel plugs were washed twice with water, twice with 50 mM ammoniumbicarbonate in 50% acetonitrile and dehydrated using 100% acetonitrile. The proteins were reduced with 10 mM DTT and subsequently alkylated with 55 mM iodoacetamide. Following washing with 50 mM ammonium bicarbonate, 50 mM ammonium bicarbonate/50% acetonitrile and 100% acetonitrile, the gel plugs were rehydrated using 50 mM ammonium bicarbonate containing 50 ng of trypsin and incubated on ice for 30 minutes. If necessary, 50 mM ammonium bicarbonate was added to completely cover the gel plugs. Protein digestion by trypsin was allowed to proceed overnight at 37°C. The supernatant was acidified using TFA to a final concentration of 0.1%, desalted over a Zip-Tip (Millipore) and spotted directly on a MALDI target plate using 1 ml α-cyano-4-hydroxycinnamic acid (HCCA, 0.3 mg/ml in ethanol:acetone 2∶1) as the Zip-Tip eluent. MS and MS/MS spectra were acquired on an Ultraflex™ II TOF/TOF instrument (Bruker Daltonics, Bremen, Germany). The MS and MS/MS spectra were searched against the IPI_Human database using the MASCOT search algorithm (version 2.1) using mass tolerances of 0.1 Da for MS and 0.5 Da for MS/MS. Carbamidomethylcysteine was taken as a fixed modification and oxidation of methionines as a variable modification. Furthermore, the search parameters allowed for 1 missed cleavage.

### 2D Western Blotting

Protein extracts were passively rehydrated into 13 cm pH 4–7 linear strips (GE Healthcare, Uppsala, Sweden) for minimal 10 hours followed by isoelectric focusing using a manifold-equipped IPGphor IEF unit (GE Healthcare, Uppsala, Sweden) according to the manufacturer's protocol. The cysteine sulfhydryls were reduced with 1.0% DTT and carbamidomethylated with 2.5% Iodoacetamide in equilibration buffer (30% glycerol, 2% SDS, 6 M urea, and 75 mM Tris, pH 8.8). Second-dimensional SDS-PAGE was performed on precast criterion Tris-HCL (12,5%) gels (Bio-Rad, Hercules, CA, USA) using the criterion ststem. The blots were blocked in hybridization buffer (10 mM Tris, 150 mM NaCl, and 0.1% Tween 20) containing 5%(w/v) milk powder (ELK, Campina, The Netherlands) for 1 h followed by incubation with first specific antibodies against (1∶1000) in hybridization buffer with 0.5% (w/v) milk powder overnight at 4°C. After incubation with the first antibody, the blots were washed five times for 3 min in hybridization buffer. The second antibody (horseradish peroxidase (HRP)-coupled; 1∶3000) was incubated in hybridization buffer containing 0.5% (w/v) milk for 2 h at room temperature followed by five washings for 3 min in hybridization buffer and a last wash step in PBS. Detection of all Western blots was performed by ECL plus (GE Healthcare, Uppsala, Sweden) and detected using a Typhoon 9410 (GE Healthcare, Uppsala, Sweden). Spot volume was analyzed using ImageQuant software (GE Healthcare, Uppsala, Sweden).

### Oxidative burst using luminol

Oxidative burst was measured in a luminometer. Isolated neutrophils were stimulated for 4 hours with different concentrations of leptin at 37°C. After 4 hours neutrophils were washed and resuspended in hanks balanced salt solution (HBSS). The luminol assay was performed in 96 wells plates containing 1×10^6^ cells/ml, 0.5% human serum albumin and luminol. The reaction was started by adding 1 mg/ml serum treated zymosan (STZ), and luminescense was measured using a FLUOstar OPTIMA, BMG Labtech (Ortenberg, Germany). Results were expressed as area under the curve.

### Statistical analysis

Data were analyzed using Graphpad Prism 5. Repeated measures ANOVA was used to compare different conditions, followed by a bonferroni's multiple comparison test comparing all conditions to non-stimulated control. A p-value <0.05 was considered significant. * p<0.05 ** p<0.005 *** p<0.001.

For proteomics statistical analysis was performed using paired Student t tests with false discovery rate correction using DeCyder 6.5 BVA. A p<0.01 was considered statistically significant. Spots had to be present in at least 70% of all spot maps.

## Results

### Leptin activity

**Figure 1:**
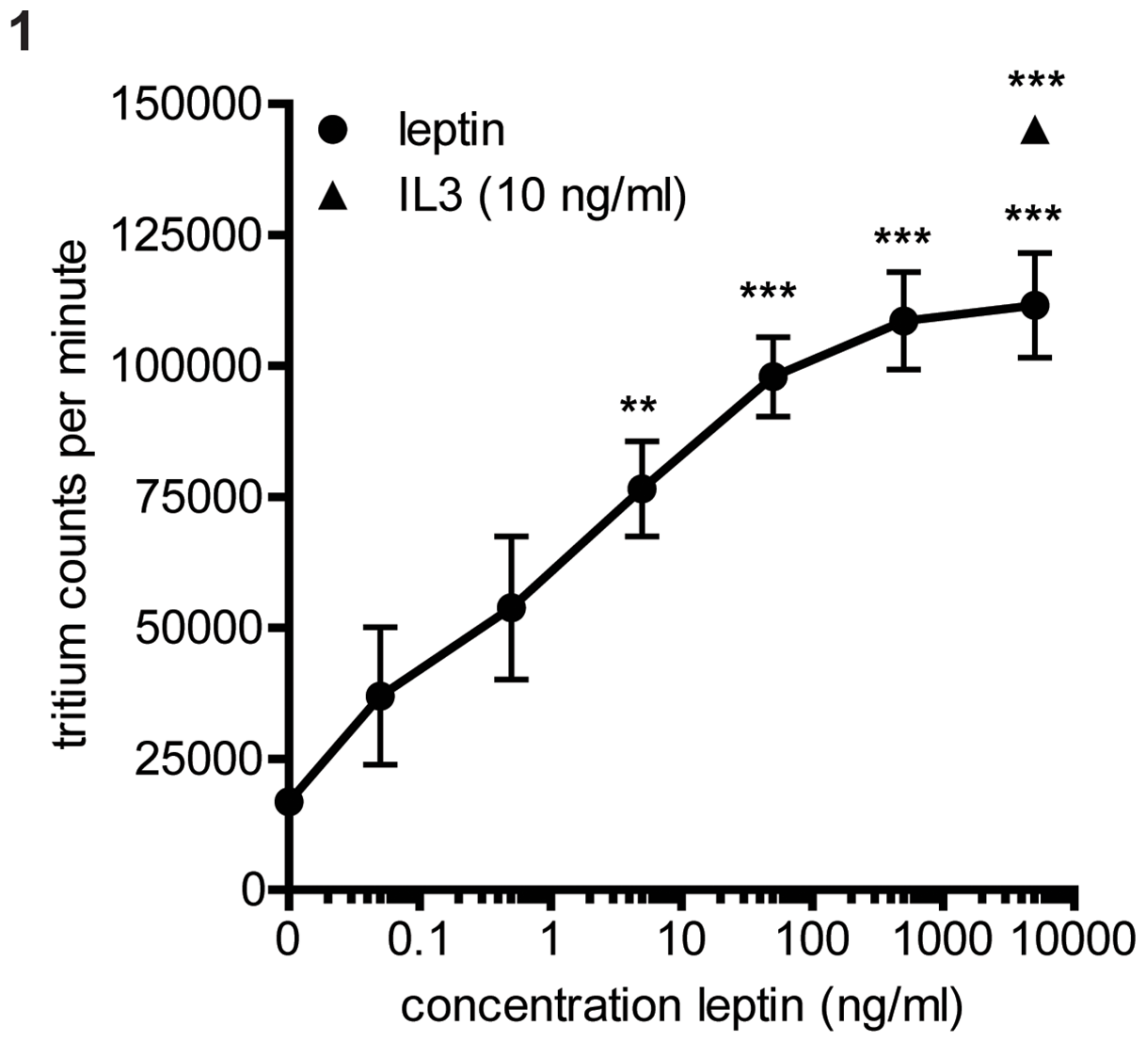
Leptin activity in OBRb Baf3 cells. Proliferation of OBRb Baf3 cells is dependent on leptin or mouse IL3. Baf3 cells were grown in medium supplemented with different concentrations of leptin or positive control IL3 (no leptin was added to the IL3 control) for 48 hours. After four days proliferation was measured by overnight radioactive tritium labeled thymidine incorporation. Graph shows the tritium counts per minute, data are expressed as mean ± SEM, n = 4. Statistics done using one-way ANOVA with Bonferroni posttest comparing leptin stimulated to control (0 ng/ml leptin), *P = <0.05, **P = <0.01, ***P = <0.001.

The bioactivity of leptin was tested using Ba/fF3 cells stably transfected with a functional human OBRb receptor [25]. Baf3 cells are cytokine dependent and normally grow in the presence of mouse IL-3. In the OBRb Baf3 cells IL-3 can be replaced by human leptin. A dose response of leptin was tested in a tritium labeled thymidine incorporation proliferation assay. Leptin activity could be detected from a concentration of 1 ng/ml onward and a plateau was reached around 1000 ng/ml (Figure 1).

### Lack of Leptin-induced activation of human neutrophils,

**Figure 2:**
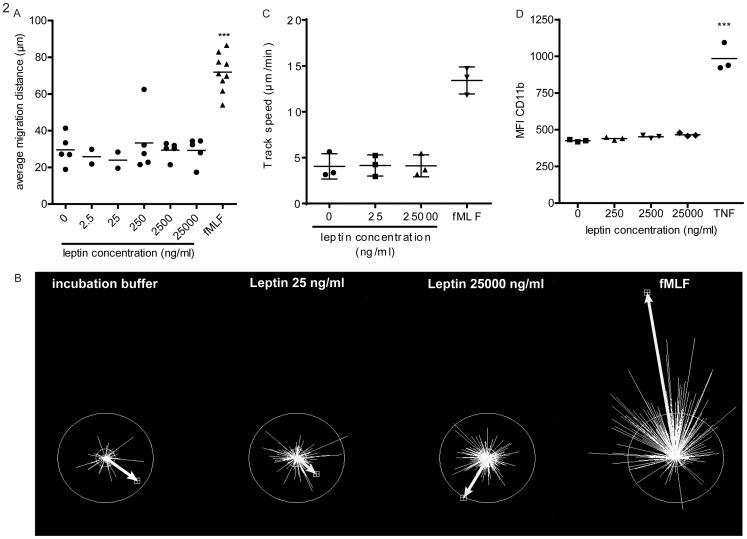
Effects of leptin on neutrophil chemotaxis and CD11b expression. (A) Isolated neutrophils were placed in a boyden chamber on top of a filter with different concentrations leptin or positive control fMLF (10^−8^ M) below the filter. Cells were allowed to migrate for 45 min at 37°C, afterwards filters were stained and average migration distance was calculated. Mean ± SEM, n = 2–9. Statistics done using one-way ANOVA with Bonferroni posttest comparing leptin stimulated to control (0 ng/ml leptin), ***P = <0.001. (B) Neutrophils chemotaxis in fibrin gel. Neutrophils were captured in a fibrin gel, chemotaxis towards leptin and positive control fMLF (10^−8^ M) in the upper compartment was recorded using a microscope system. Afterwards vector tracks of all the cells were centered and directionality was determined. Directionality is shown by the white arrow, and is significant when the arrow reaches outside the circle. (C) Chemokinesis in a fibrin gel. Again neutrophils were captured in a fibrin gel but for the chemokinesis experiments the leptin or positive control fMLF (10^−8^ M) was present throughout the gel. Migration in the gel was recorded using a microscope system and track speed was analyzed. (D) Neutrophils were isolated and incubated with different concentrations of leptin or TNF (100 U/ml) as positive control for four hours. Afterwards cells were stained for CD11b, and mean fluorescence intensity (MFI) was determined by flow cytometry. Data are expressed as mean ± SEM, n = 3. Statistics are done using one-way ANOVA with Bonferroni posttest comparing leptin stimulated to control (0 ng/ml leptin).

Not many studies have shown direct effects of leptin on the functionality of neutrophils. However, several studies have suggested that leptin is a chemoattractant for neutrophils [16], [17]. Ottonello et al have shown that Leptin is chemotactic for human neutrophils with a maximum migration at a concentration of 50 ng/ml [16], [17]. We attempted to reproduce these results. However, leptin did not induce chemotaxis in our experiments applying classical Boyden chambers, whereas the positive control fMLF was active at the expected concentration of 10 nM [26] (Figure 2A). A wide range of leptin concentrations was tested, ranging from 2.5 ng/ml up to 25.000 ng/ml, but none of the concentrations led to any chemotaxis (Figure 2A). Additionally a different chemotaxis assay using real time imaging of migrating neutrophils in fibrin gels did not show any chemotaxis (Figure 2B, representative of three separate experiments). In this experiment the arrow shows both directionality as well as statistical significance. Leptin or the positive control was present in the upper compartment. The directionality was only significant when the arrow is outside the circle. Only fMLF showed significant chemotaxis in response to the gradient, whereas no leptin-induced response was detected.

As both of our chemotaxis assays did not show any chemotaxis of neutrophils towards leptin we tested the hypothesis that Ottonello et al might have detected chemokinesis (random movement) rather than chemotaxis (directed movement. These chemokinesis experiments were performed in similar fibrin gels. There was no chemokinesis detectable upon leptin stimulation (Figure 2C).

Apart from chemotaxis and chemokinesis other neutrophil responses were evaluated such as upregulation of CD11b. Upon neutrophil activation secretory vesicles fuse with the plasma membrane, thereby increasing the CD11b expression [27]. Leptin did not increase CD11b expression, whereas the positive control TNFα did (Figure 2D). Effects on several other receptors was tested (CD62L, CBRM1/5 the active conformation of CD11b, CD66b, CD88, CD181, CD182, and CD16), but none of these receptors changed expression upon leptin stimulation (Figure S1).

### Leptin does not induce changes in the neutrophil proteome

To study whether leptin has another effect on neutrophils an unbiased 2D-DIGE proteomics approach was used. A high leptin concentration (250 ng/ml  = 8 times the average concentration found in obese people, though concentrations around 100 ng/ml are found in some obese subjects [21]), was chosen to evaluate putative changes in neutrophil proteome. Neutrophils were stimulated for 4 hours. After stimulation cells were lysed and proteins were analyzed using 2D gel electrophoresis. DeCyder software was used to analyze the results. Spots were marked as different when there ratio between unstimulated and stimulated was <−1.3 or >1.3 and the T-test P-value was smaller than 0.01. Statistical analysis showed no significant differences between control and 250 ng/ml leptin (data not shown).

### Leptin-induced neutrophil survival

**Figure 3:**
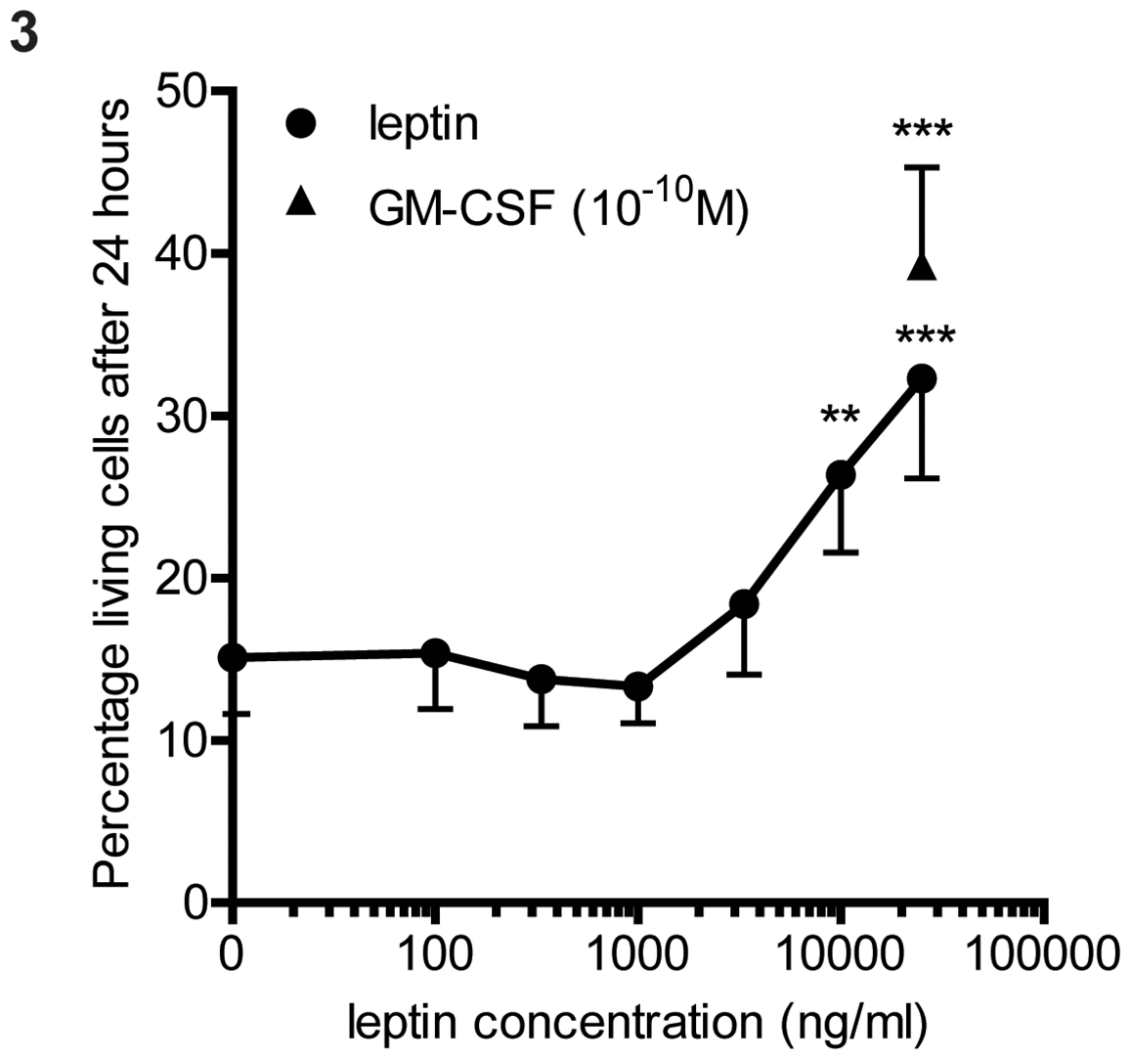
Effects of leptin on neutrophil survival. Neutrophils were isolated and incubated with different concentrations of leptin or GM-CSF as positive control. Cells were stained for Annexin V after 24 hours. The percentage annexin negative cells is the percentage lving cells. Data are expressed as mean ± SEM, n = 5. Statistics done using one-way ANOVA with Bonferroni posttest comparing leptin stimulated to control (0 ng/ml leptin), **P = <0.01, ***P = <0.001.

Another characteristic attributed to leptin is induction of neutrophil survival [20]. However, no appreciable survival could be induced by physiologically relevant concentrations of leptin (1–100 ng/ml see also Figure 1). At extremely high concentrations leptin could induce neutrophil survival (Figure 3). Leptin induced neutrophil survival started around 3000 ng/ml, but the plateau was still not reached at a concentration of 25000 ng/ml (Figure 3).

### High leptin concentrations induced changes in neutrophil proteome

**Figure 4:**
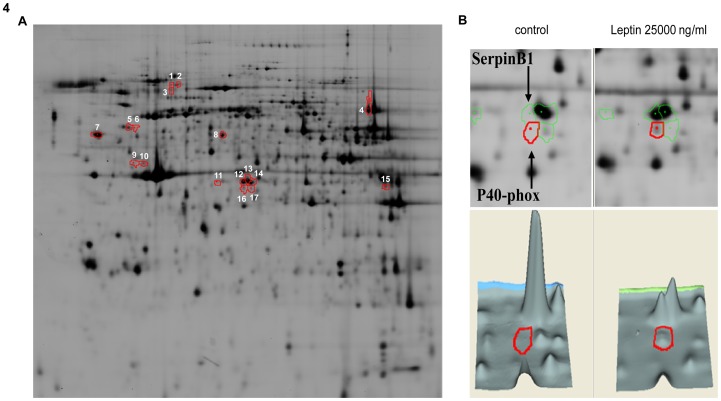
Proteome analysis upon leptin stimulation. Neutrophils were stimulated for four(IEF strip pH 3–10 nonlinear 24 cm). (A) Representative example of a 2D gel showing all protein spots chosen for mass-spectrometry analysis. (B) Example of two proteins that shift in pI upon leptin stimulation, SerpinB1 and p40-phox.

No differences could be detected in the neutrophil proteome after stimulation with 250 ng/ml leptin. Leptin dependent neutrophil survival was, however, initiated at much higher concentrations. Therefore, the same unbiased 2D-DIGE proteomics approach as described above was used to test the effects of 25000 ng/ml leptin on the neutrophil proteome. Again spots were marked different when there ratio between unstimulated and stimulated was <−1.3 or >1.3 and the T-test P-value was smaller than 0.01. Between control and 25000 ng/ml leptin stimulation twelve differently regulated spots could be detected (Table 1). These 12 spots were chosen for identification by mass-spectometry. To increase the change of identification adjacent spots, which probably consist of the same protein, these proteins were also selected for identification (indicated in Table 1 by^1^). The positions of the spots sent for mass-spectrometry analysis were indicated in a representative 2D gel (Figure 4A). The identified spots were picked for mass spectrometry analysis, and thirteen were identified (Table 1).

**Table 1:** Spots differently regulated upon leptin (25000 ng/ml) stimulation.

Spot nr.	name	T test	Ratio	Swiss-Prot	Theoretical Mass (kDa)	Theoretical pI
**1**	Myosin-9 isoform 1	5.21E-04	−2.23	P35579-1	227.0	5.5
**2**	Myosin-9 isoform 1	1.83E-03	−1.48	P35579-1	227.0	5.5
**3**	Not Identified	4.52E-01	−1.14			
**4**	Transketolase isoform 2	8.70E-03	1.34	P29401	67.9	7.6
**5**	cDNA FLJ52902 highly similar to Rab GDI alpha^1^	2.01E-01	−1.15	B4DHX4	46.9	4.9
**6**	Not Identified	3.58E-03	−1.39			
**7**	Protein disulfide-isomerase	6.22E-03	−1.43	P07237	57.1	4.8
**8**	Protein disulfide-isomerase A3	4.08E-03	−1.33	P30101	56.8	6.0 / 5.5
**9**	Thioredoxin domain-containing protein 4	6.56E-03	−1.73	Q9BS26	47.0	5.1
**10**	Not Identified	4.33E-03	−1.75			
**11**	Actin cytoplasmic 2	1.06E-02	−2.38	P63261	41.8	5,4
**12**	Serpin B1^1^	2.36E-02	3.16	P30740	42.7	5.9
**13**	Serpin B1	6.13E-04	−1.49	P30740	42.7	5.9
**14**	Gelsolin like capping protein^1^		−1.17	P40121	38.5	5.9
**15**	Sorbitol dehydrogenase	2.62E-03	2.02	Q00796	38.3	8.2
**16**	p40 phox^1^	1.59E-02	3.28	Q15080-2	39.0	9.1
**17**	Not Identified^1^	2.01E-02	−3.13			
						

1 Spots were picked because they were next to a spot that was differently regulated.

SerpinB1 was chosen for further investigation, as this protein is known to be important for protection from neutrophil elastase [28]. Additionally p40-phox which is part of the NADPH oxidase complex was chosen, because this protein was identified in the same cluster of spots as SerpinB1 and is a regulator of the neutrophil respiratory burst [29]. Both proteins showed a shift to the left upon leptin stimulation (Figure 4B). Phosphorylation results in a left shift in isoelectric point and this shift can, therefore, be an indication of increased phosphorylation.

### No differences in p40 phox expression induced by leptin detected by western blot

**Figure 5:**
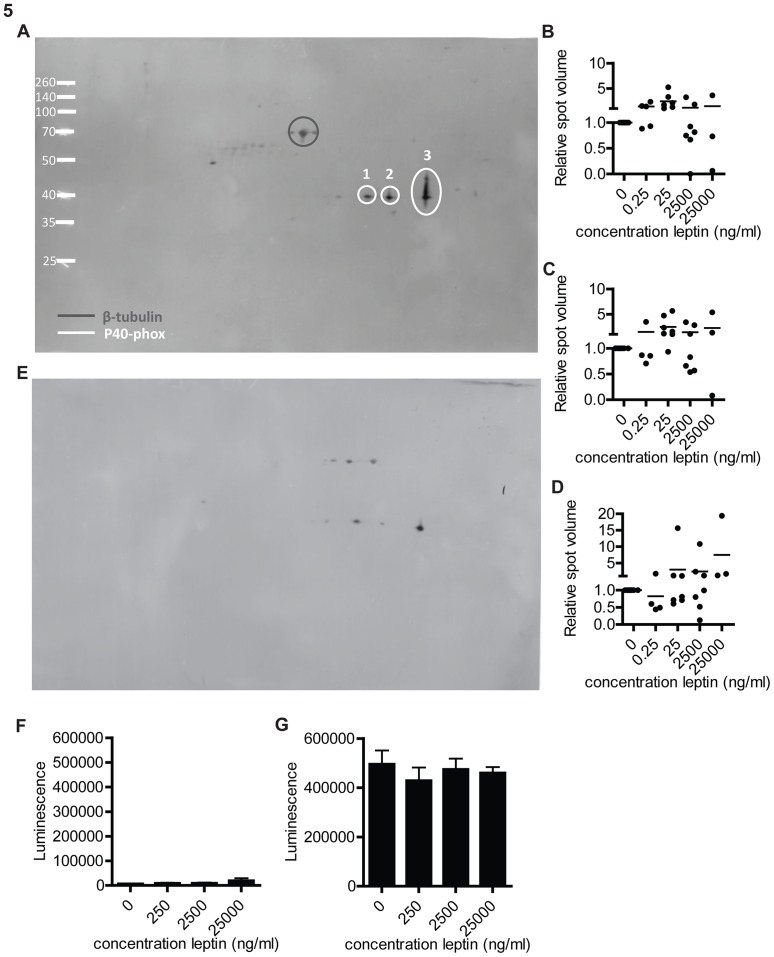
Westernblot analysis of p40 phox after leptin stimulation. Neutrophils were stimulated with different concentrations of leptin for four(IEF strip pH 4–7 linear 13 cm) and 2D gels were blotted. (A) Representative example of a 2D westernblot stained for p40 phox and β-tubulin. (B–D) Quantification of spot volume corrected for β-tubulin and normalized against the 0 ng/ml leptin control, for p40 phox spot 1 (B), spot 2 (C) and spot 3 (D). Data are expressed as mean ± SEM, n = 3–8. Statistics done using one-way ANOVA with Bonferroni posttest comparing leptin stimulated to control (0 ng/ml leptin). (E) Representative example of a 2D westernblot stained for phosphorylated p40-phox (F,G) Intracellular luminol luminescence was measured in the context of different concentration of leptin. Neutrophils were not stimulated (E) or stimulated with STZ (F). data are expressed as mean ± SEM, n = 4–5. Statistics done using one-way ANOVA with Bonferroni posttest comparing leptin stimulated to control.

To study the differences found in p40phox isoelectric point (pI) in more detail western blots were performed. As shifts in pI cannot be confirmed by normal western blots, small 2D gels were run and blotted. To get sufficient separation between the different isoelectric points on this smaller surface, IEF strips pH 4–7 linear (13 cm) were used instead of the 3–10 non-linear (24 cm) IEF strips used in the 2D-DIGE experiments. The disadvantage of this 2D blotting is that every sample needs its own blot, complicating the comparison between samples. β-tubulin was used as a control protein enabling us to correct for loading differences. A representative example of a 2D western blot is shown (Figure 5A). White circles indicate the p40-phox spots and grey circles β-tubulin protein spots. No effects were seen upon leptin stimulation (Figure 5B–D), all spots had a similar volume before and after leptin stimulation. Next to a total p40 phox antibody an antibody against phosphorylated p40 phox (Thr154) was used for detection, to our surprise this antibody stained the same spots as the total p40 phox antibody, plus some additional spots (Figure 5E). This suggested that all p40 phox proteins found at these spots were phosphorylated at this specific site (Thr154).

### Absence of leptin induced activation of the intracellular respiratory burst

The molecule p40phox has been shown to be important for intracellular Reactive oxygen species (ROS) production in a p40phox deficient patient [29]. Therefore, intracellular ROS production was tested in leptin stimulated cells using luminol. STZ was used as stimulation for intracellular ROS and extracellular ROS detection was quenched by HAS [30]. Leptin did not effect this intracellular ROS production in control (Figure 5E) or fMLF stimulated cells (Figure 5C).

### No leptin induced differences in SerpinB1 expression detected by western blot

**Figure 6:**
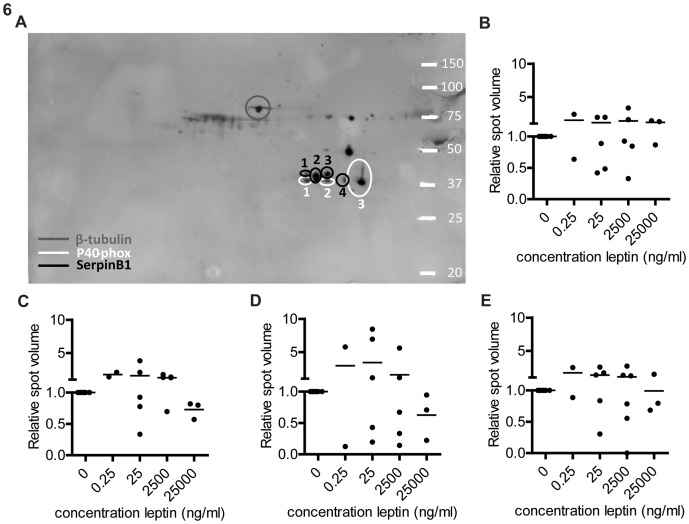
Westernblot analysis of SerpinB1 after leptin stimulation. Neutrophils were stimulated with different concentrations of leptin for four(IEF strip pH 4–7 linear 13 cm) and 2D gels were blotted. (A) Representative example of a 2D westernblot stained for SerpinB1, p40 phox and β-tubulin. (B–E) Quantification of spot volume corrected for β-tubulin and normalized against the unstimulated control, for SerpinB1 spot 1 (B), spot 2 (C), spot 3 (D) and spot 4 (E). Data are expressed as mean ± SEM, n = 2–6. Statistics done using one-way ANOVA with Bonferroni posttest comparing leptin stimulated to control.

The protein identified as serpinB1 in the 2D-DIGE experiments showed a similar shift in pI as p40 phox. Therefore, blots used for p40 phox analyses were co-stained for SerpinB1 (Figure 5A), spot volumes were calculated and corrected for β-tubulin. There were no significant differences in spot volume upon leptin stimulation (Figure 6B–E).

## Discussion

This study has been set out to test the hypothesis that leptin is a true neutrophil agonist. This is important knowledge as a body of literature suggest that this adipokine is important in (systemic) inflammation in obese individuals [1]. Particularly, aberrant systemic activation of neutrophils is thought to play an important role in low grade inflammation [31]. In marked contrast to these studies that were mainly based on association between leptin and indicators of disease in obesity, surprisingly little is known regarding the leptin receptor on neutrophils and the functional responsiveness of these cells to this adipokine.

A small number of studies have shown that leptin is a potent chemoattractant for human neutrophils^15,16^. However, we could not reproduce these data as leptin did not attract neutrophils in our chemotaxis experiments, while leptin was biologically active as it induced proliferation of Baf3 OBRb cells. Our results contradict the earlier results [16], [17], while similar methods for detecting chemotaxis were used. The only important difference between our assays and that of Otonello and Montecucco was that the latter applied the the leading front method, measuring the migration distance from the fastest cells in the filter. Our results on the other hand, shown in Figure 2A, were determined by measuring the mean migration distance of all cells migrated into the filter [32]. The leading front method can be biased by a few cells that migrate very fast [33]. A small contamination with monocytes, which are known to migrate upon leptin stimulation [34], could therefore lead to bias in the leading front assay. Additionally all true chemoattractant receptors on neutrophils belong to the family G-protein coupled serpentine receptors [35], while the leptin receptor belongs to the family of cytokine receptors. Ligands that induce migration through non-serpentine receptors evoke chemokinesis rather than chemotaxis [24], [35]. Neither chemotaxis nor chemokinesis upon leptin stimulation was detected in a microscopic chemotaxis assay.

If leptin is a neutrophil ligand, expression of a signaling competent leptin receptor is required There is, however, debate whether neutrophils express the long signaling form of the leptin receptor, OBRb [15], [18]. We could not detect any expression of this OBRb receptor on human neutrophils by quantitative PCR (data not shown), which is known to activate the JAK/STAT signaling pathway in other cells [3], [36]. The leptin receptor that is likely expressed on neutrophils, OBRa [15], [18], has been described to signal via the MAPK/ERK pathway in COS cells, but to a lesser extent than the OBRb receptor [37]. In contrast, others have suggested that OBRa can only signal in a Jak2 overexpression system [3]. In agreement with this latter study, transfection of Baf3 cells with the OBRa receptor did not result in leptin dependent proliferation [25]. We decided to use an unbiased proteomics approach to see whether leptin induced signals in neutrophils when stimulated with leptin in a concentration near physiological concentrations (250 ng/ml). But no significant changes were detected. Therefore, we find it unlikely that neutrophils express a signaling leptin receptor OBRb or OBRa [15], [18] that could transduce any detectable signals when near physiological concentration were used.

Neutrophil apoptosis was also found to be inhibited at very high leptin concentrations [20]. These results were confirmed by our experiments. The underlying mechanism is yet to be defined but the exceptionally high concentrations of leptin required for this effect suggest that it is an indirect effect. Still, we decided to study the putative changes in neutrophil proteome at this high leptin concentration. Leptin induced differences in the neutrophil proteome when high concentrations were used. Nonetheless the high concentrations necessary to induce neutrophil survival and changes in the proteome raised the question of in vivo relevance. Even the high leptin concentrations found in obese patients (31.3±24.1 ng/ml) [21] are far below the concentrations needed for these effects (3000–25000 ng/ml). Additionally, it can be argued whether these are indirect effects rather than a leptin receptor mediated effects. Although this issue is difficult to address it is clear that survival of neutrophils is greatly influenced by the environment. Different proteins coated on the plastic surface of the culture flask can have marked effects of neutrophil survival by e.g. preventing the aspecific activation of the NADPH-oxidase [38], [39]. The underlying protecting effect of the environment on neutrophil functionality has not been elucidated. It can, therefore, not be ruled out that most of the leptin induced effects are caused by such an indirect protective effect preventing culture induced acceleration of neutrophil apoptosis and aspecific activation.

Also the leptin induced effects on the neutrophil proteome might have been indirect effects of leptin. As both activation of the respiratory burst and degranulation of e.g. proteases are also affected by adhesion tot surfaces. The differences between expression of P40 phox and and SerpinB1 analyzed by 2D-DIGE and western blot are difficult to explain, but their expressions are characterized by multiple spots in the 2D gels. It is not clear what the differences are between the spots and whether the blotting antibodies recognize all spots equally well. In addition, when leptin induced changes are indirect and mediated by unknown mechanisms, it is difficult to predict how subtle changes in experimental design will affect the results.

In conclusion, we found no convincing evidence that leptin is a neutrophil agonist acting through a functional leptin receptor on neutrophils. Therefore, it is likely that all in vivo effects of leptin on neutrophil functionality are indirect as was previously suggested [14], [15], [40].

## Supporting Information

Figure S1 **Effects of leptin on different neutrophil activation receptors.** Neutrophils were isolated and incubated with different concentrations of leptin or TNF (100 U/ml) as positive control for four hours. Afterwards cells were stained for (A) CD62L, (B) CBRM1/5 recognizing the active conformation of CD11b, (C) CD66b, (D) CD88, (E) CD181, (F) CD182 and (G) CD16. Mean fluorescence intensity (MFI) was determined by flow cytometry. Data are expressed as mean ± SD, n = 3.
(TIF)
