# Supplementary figures and images for: Physiological Concentrations of Leptin Do Not Affect Human Neutrophils
Source: PLoS One. 2013 Sep 16;8(9):e73170. doi: 10.1371/journal.pone.0073170 (PMC3774682; doi:10.1371/journal.pone.0073170)

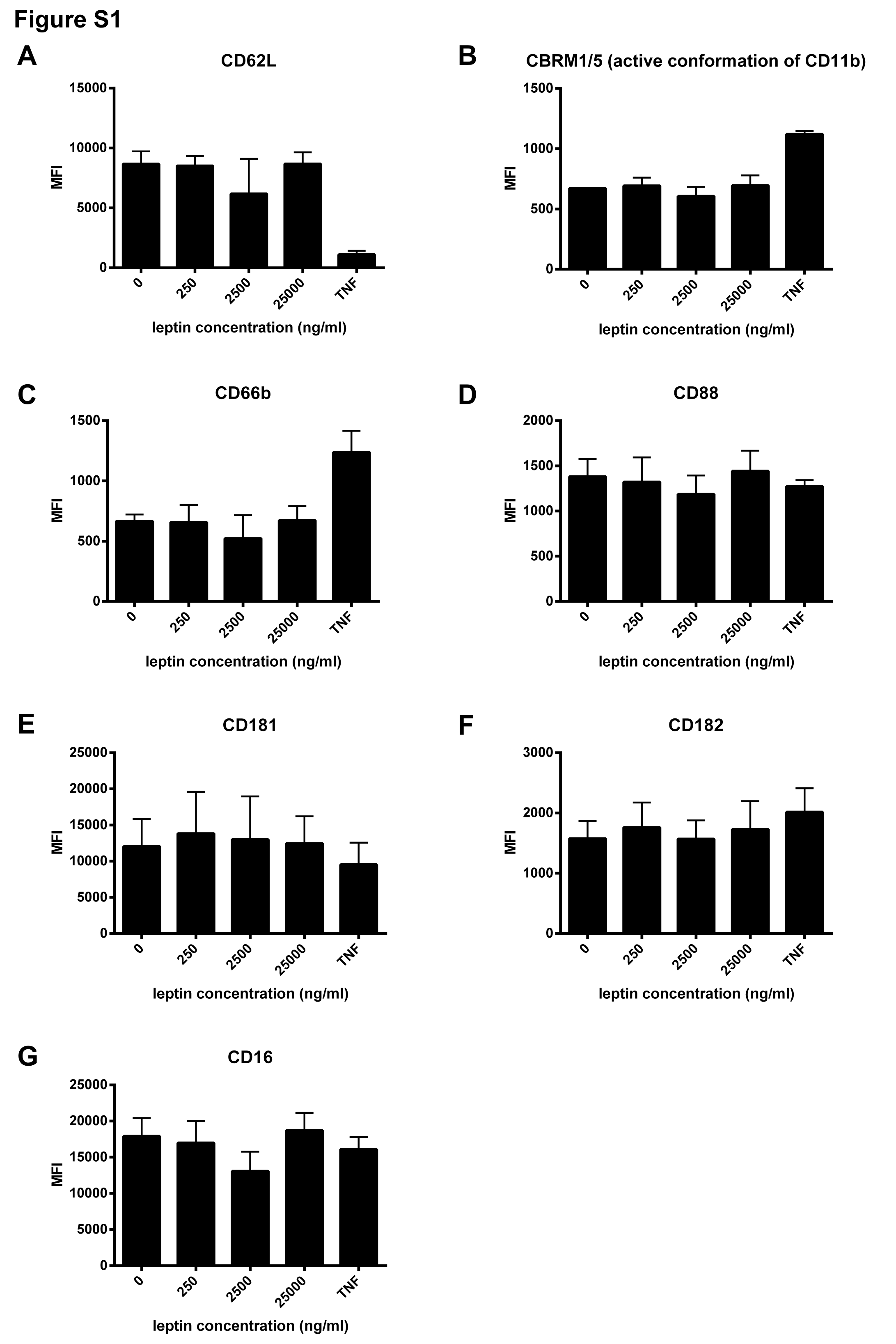

Supplement: Figure S1 — Effects of leptin on different neutrophil activation receptors. Neutrophils were isolated and incubated with different concentrations of leptin or TNF (100 U/ml) as positive control for four hours. Afterwards cells were stained for (A) CD62L, (B) CBRM1/5 recognizing the active conformation of CD11b, (C) CD66b, (D) CD88, (E) CD181, (F) CD182 and (G) CD16. Mean fluorescence intensity (MFI) was determined by flow cytometry. Data are expressed as mean ± SD, n = 3. (TIF) [file pone.0073170.s001.tif]
